# Occipital and Cingulate Hypometabolism are Significantly Under-Reported on 18-Fluorodeoxyglucose Positron Emission Tomography Scans of Patients with Lewy Body Dementia

**DOI:** 10.4172/2161-0460.1000428

**Published:** 2018-02-28

**Authors:** Moath Hamed, Frank Schraml, Jeffrey Wilson, James Galvin, Marwan N Sabbagh

**Affiliations:** 1 Alzheimer’s and Memory Disorders Division, Department of Neurology and Nuclear Medicine, Barrow Neurological Institute, USA; 2 Barrett Honors College and W. P. Carey School of Business, University of Arizona State, USA; 3 Schmidt College of Medicine,University of Florida Atlantic, Boca Raton, FL, USA; 4 Cleveland Clinic Lou Ruvo Center for Brain Health, Las Vegas NV, USA

**Keywords:** Lewy body dementia, Visual cortices, Hypometabolism

## Abstract

**Objective:**

To determine whether occipital and cingulate hypometabolism is being under-reported or missed on 18-fluorodeoxyglucose positron emission tomography (FDG-PET) CT scans in patients with Dementia with Lewy Bodies (DLB).

**Background:**

Recent studies have reported higher sensitivity and specificity for occipital and cingulate hypometabolism on FDG-PET of DLB patients.

**Methods:**

This retrospective chart review looked at regions of interest (ROI’s) in FDG-PET CT scan reports in 35 consecutive patients with a clinical diagnosis of probable, possible, or definite DLB as defined by the latest DLB Consortium Report. ROI’s consisting of glucose hypometabolism in frontal, parietal, temporal, occipital, and cingulate areas were tabulated and charted separately by the authors from the reports. A blinded Nuclear medicine physician read the images independently and marked ROI’s separately. A Cohen’s Kappa coefficient statistic was calculated to determine agreement between the reports and the blinded reads.

**Results:**

On the radiology reports, 25.71% and 17.14% of patients reported occipital and cingulate hypometabolism respectively. Independent reads demonstrated significant disagreement with the proportion of occipital and cingulate hypometabolism being reported on initial reads: 91.43% and 85.71% respectively. Cohen’s Kappa statistic determinations demonstrated significant agreement only with parietal hypometabolism (p<0.05).

**Conclusion:**

Occipital and cingulate hypometabolism is under-reported and missed frequently on clinical interpretations of FDG-PET scans of patients with DLB, but the frequency of hypometabolism is even higher than previously reported. Further studies with more statistical power and receiver operating characteristic analyses are needed to delineate the sensitivity and specificity of these *in vivo* biomarkers.

## Introduction

Dementia with Lewy Bodies (DLB) is the second-most common cause of dementia in North America behind Alzheimer’s disease, and is characterized by cognitive, motor, and neuropsychiatric disturbances that usually follow a fluctuating, progressive course [1]. While the latest McKeith Consensus Criteria possesses high specificity for DLB patients, 18-fluorodeoxyglucose positron emission tomography, or FDG-PET, has been touted as a reliable modality for diagnosing DLB by way of occipital hypometabolism in patients with dementia [2,3]. Albin et al. [4] looked at 6 patients with pathologically-confirmed DLB and found significant occipital and primary visual cortex hypometabolism. Ishii et al. [5] investigated 36 patients with clinical DLB and found significant p-values for cerebral glucose metabolic rate (CMRglc) values in occipital lobes compared to patterns seen in Alzheimer’s Disease and normal controls.

Minoshima et al. found that antemortem occipital hypometabolism on FDG-PET had 90% sensitivity and 80% specificity for DLB [6]. The largest multi-center trial to date, one led by Mosconi et al. [7] looked at a total of 548 patients including both healthy controls and dementia patients, 27 of them carrying a definite DLB diagnosis as defined by the McKeith Consensus Criteria, and found that occipital hypometabolism had a 71% specificity for DLB. O’Brien et al. found that occipital hypometabolism had a 64% positive predictor value for the disease [8]. Fujishiro et al. [9] studied patients with occipital hypometabolism regardless of comorbid dementia and found that FDG-PET findings in the primary visual cortex can be associated with conditions other than DLB such as REM-sleep behavioral disorder and Parkinson’s Disease.

These findings cast doubt to the overall reliability of occipital hypometabolism on its own as a biomarker for dementia with Lewy bodies. Other regions of interest have been identified in DLB, such as preservation of posterior cingulate island perfusion, which can enhance specificity for DLB on FDG-PET studies up to 100% when taken alongside occipital hypometabolism concurrently [10]. Prosser et al. [11] found that preserved cingulate island perfusion and reduced occipital hypometabolism on Tc-HMPAO SPECT increase specificity but not sensitivity for DLB. Conversely, another study by Graff-Radford et al. [12] found that the degree of cingulate island metabolism was inversely correlated with Braak Neurofibrilary Tangle (NFT) staging, suggesting coexistence of Alzheimer Disease pathology in patients carrying a diagnosis of probable DLB and exhibiting cingulate hypoperfusion. It is our hypothesis that occipital hypometabolism on FDG-PET is under-reported and often missed on radiological reports. Our objective was to investigate the degree of inter-reporter agreement between radiology reports and independent radiological reads of FDG-PET scans regarding hypometabolism in major cerebral regions of interest (ROI’s), with focus on occipital and cingulate metabolism.

## Methods

### Approval of human subject protocol

The study was conducted according to the human subject study protocol approved by Barrow Neurological Institute (BNI) Institutional Review Board. Because this was a retrospective chart review with minimal inherent risk to patients, expedited approval was granted. Informed consent was waived as this retrospective cohort study did not involve direct patient participation, obtaining informed consent was time-consuming and unnecessary given minimal risk to patient privacy, and the records were studied under protocols enforced by our organization’s Institutional Review Board.

### Subjects

Outpatient electronic medical record database was queried for all patients carrying ICD-9 code diagnosis 331.82 or ICD-10 code diagnosis G31.83, namely Dementia with Lewy Bodies, who have had diagnostic FDG-PET scans within the last three years [13]. Thirty-nine consecutive patients with a clinical diagnosis of DLB who had had an FDG-PET of the brain acquired during the diagnostic evaluation were included. Four patients with non-diagnostic scans were excluded from the study. Patients with a clinical diagnosis of DLB who did not undergo a FDG-PET were excluded. Outpatient visit records were then filtered manually for features of the disease in accordance with the Fourth McKeith Consensus Criteria published in 2017 [2]. In this manner, thirty-five patients carrying a clinical diagnosis of possible, probable, and definitive Dementia with Lewy Bodies were included in this study (Table 1). Subjects with alternative dementia diagnoses – Parkinson’s Disease Dementia, Alzheimer’s Disease, and Frontotemporal Dementia -and patients exhibiting secondary features of delirium akin to DLB (hallucinations, fluctuating awareness) were excluded from this study. Healthy controls were not used in this study. Demographics of the patients described in the above section, namely age, gender, age at diagnosis, disease duration, and core clinical features, were compiled and tabulated.

[18F]-Fluorodeoxyglucose (FDG) PET acquisition and pre-processing: FDG-PET was used to compare regional cerebral glucose metabolism across groups as described in previous literature [10].

### Neuroradiology/nuclear medicine FDG-PET analysis

The clinical reports were gathered with review of the impression and technical aspects. The data-gathering portion of the study involved reviewing radiological reports and tabulating hypometabolism in regions of interest (ROI’s)-frontal, temporal, parietal, occipital, and cingulate as either present or absent (1 or 0 respectively). The analytical portion of the study involved a single-blinded nuclear medicine physician (FS) read of FDG-PET scans of the patients compiled in the abovementioned manner in the descriptive portion of the study. All patients queried in the above manner with probable or possible DLB had their FDG-PET scans read by a blinded Nuclear Medicine physician who determined regional hypometabolism. The Nuclear medicine physician marked regions of interest (ROI’s) exhibiting hypometabolism based on 0-1 scale of either absent or present respectively. Primary visual and visual association cortical metabolism was also investigated.

### Statistical analysis

The proportions of hypometabolism for each region of interest (frontal, temporal, parietal, occipital, and cingulate) in both the radiological and independent reads were calculated by dividing the number of positive scans for that ROI by the total number of patients. These proportions were compared side-by-side, and a Cohen’s kappa coefficient was calculated to assess for independent reader agreeability with the prior reported regions of hypometabolism [14].

## Results

The demographic characteristics, clinical diagnoses, and functional outcome measures are shown in (Table 1). The sample included 35 patients (23M, 12F) with a mean age of 73.77 years and disease duration of 3.71 years at the time of the FDG-PET scans 94.3%, exhibited fluctuating cognition, while Parkinsonism and visual hallucinations were present in 88.6% and 57.1% respectively. Seventeen (17) patients, comprising 48.57% of our patient population, carried a diagnosis of definite DLB (three of four core clinical features as defined by McKeith Consensus Criteria, 2017), while 16 (45.71%) patients carried a diagnosis of probable DLB (two of the four core clinical features). Only 2 (5.71%) patients had one of the core clinical features to classify as possible DLB. Table 2 demonstrates the percent of scans with reported hypometabolism by region of interest in both the radiology reports and the independent nuclear medicine physician blinded read. Temporal and parietal hypometabolism were found in 88.6% and 91.43% of patients respectively, whereas frontal hypometabolism was found in only 37.1% of patients on clinical reports. Occipital hypometabolism was reported in 25.7% of cases, while cingulate hypometabolism was reported in 17.1% of cases.

On independent reads by the nuclear medicine physician, similar proportions of regional hypometabolism were tabulated for frontal, temporal, and parietal hypometabolism. Discrepancies in proportion existed in occipital and cingulate hypometabolic reads between clinical reports and nuclear medicine reads blinded to the clinical reads, with 32 and 30 (91.43% and 85.71%) patients out of 35 exhibiting occipital and cingulate hypometabolism respectively. 91.43% exhibited hypometabolism in the visual association cortex and 11/32 (34.38%) in the primary visual cortex. Only one patient had unilateral occipital hypometabolism, whereas the majority with occipital hypometabolism had it bilaterally. Table 3 demonstrates that even among the definitive DLB subgroup of patients in this study (n=17), only 23.53% were found to have occipital hypometabolism reported on radiology reports. The prevalence of reported occipital hypometabolism among the probable and possible subgroups of DLB patients was found to be 25% and 50% respectively. Independent reads demonstrated significant disagreement with the proportion of occipital hypometabolism, and differences existed among definite, possible, and probable DLB subgroups as well. Disagreement also existed with the proportion of frontal, temporal, and, interestingly, cingulate hypometabolism between the reports and the independent reads. Cohen’s Kappa statistic determinations demonstrated only significant agreement with parietal hypometabolism (p<0.05) but not with occipital or cingulate (p=0.287 and 0.272>0.05 (Table 4).

## Discussion

The significance of this study is that occipital hypometabolism as an *in vivo* biomarker was found to be grossly under-reported on clinical reports. Further, we find that 91.43% of patients exhibit occipital hypometabolism, whether in the primary visual or visual association cortices, compared to 25.71% on prior radiological reports. Significant disagreement existed between radiology reports and our independent reads, with a Cohen’s kappa coefficient 0.063 and a p-value of 0.282>0.05. The novelty is that these data suggest the occipital hypometabolism might be missed clinically but the frequency is actually higher than previously reported [8].

Literature surrounding occipital hypometabolism in Dementia with Lewy Bodies share certain discrepancies in reporting specifically the nature of occipital hypometabolism. Some authors indicate that the hypometabolism is localized to the primary visual cortex, whereas other studies indicate that the visual association cortex is primarily affected [4,15]. Concerning is the absence of such mention in the latest DLB Consortium Consensus Report, where occipital hypometabolism with or without the cingulate island sign on FDG-PET has been touted a supportive biomarker for diagnosing DLB [2]. Earlier studies did not specify where in the occipital lobe the hypometabolism existed. Whether eyes are open or closed during uptake studies were not specified in some studies regarding their subjects, although the most cited studies do make such mention [10].

One recent study found that hypometabolism in the visual association cortices is probably a more sensitive indicator of Lewy body disease while hypometabolism in the primary visual cortex is probably more specific [15]. Our data appears to support this conclusion, with the majority of DLB patients in our study exhibiting visual association cortex hypometabolism. However, differences might be partly due to the differences between visual reads versus automated reads by SUVR. Interestingly, cingulate hypometabolism was also found to be missed frequently in patients meeting clinical criteria for DLB, and significant disagreement existed between radiological reports and independent reads. Classically, the cingulate island sign, identified as sparing of metabolism in the posterior cingulate region as compared to the precuneus and cuneus, was identified as a specific marker when taken alongside occipital hypometabolism [11,12]. Other studies found that the degree of cingulate hypometabolism in the presence of occipital hypometabolism may correlate to coexisting AD pathology as well [12].

From these data, it is possible to speculate that when a general radiologist rather than a nuclear medicine specialist is reading PET scans for a local hospital, there will likely be differences. It is common practice for nuclear medicine, Neuroradiologists, and general radiologists to read a wide variety of scans. This could account for the heterogeneity in detection of hypometabolic changes. This study is not without its limitations. The first is that core clinical criteria were used in the inclusion of patients into this study, and as such DLB was considered to the primary diagnosis for these patients at the time of FDG-PET scanning. Therefore, coexisting pathology was not taken into consideration. That being said, the second limitation is that probable DLB carries with it a high proportion of coexisting Alzheimer’s Dementia pathology on previous studies, and this study did not specifically address the distinction between DLB and mixed Lewy Body and Alzheimer pathologies as evidenced on cingulate hypometabolism. However, clinical features strongly suggested a predominantly Lewy Body Dementia picture for most patients. The third is that although 90% of patients had their eyes open during the study as per protocol, around 2 had their eyes closed per the technician and a handful had indeterminate eye opening/closure status. This influences the scan by causing hypometabolism in the occipital lobe, which can confound the diagnostic picture [16].

**Figure 1:**
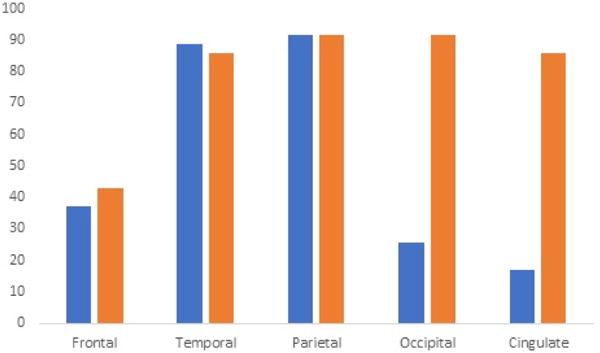
Proportion of hypometabolism in regions of interest on radiology reports (blue) compared to independent reads (orange) expressed as percentage (%).

However, the proportion of patients with eyes closed during the scan was felt to be negligible in this study. Another potential limitation is selection bias, since the primary selection apart from diagnosis is whether they had had FDG-PET which was largely determined by whether the scans were underwritten by insurance companies. The primary objective of this study is to demonstrate the under-reporting of occipital hypometabolism by identifying patients with occipital hypometabolism per radiologist reports, and determining the agreeability of such a read with an independent, blinded Nuclear Medicine physician. Occipital hypometabolism is therefore not reliably reported and often missed. These findings could establish a cohort for longitudinal research of FDG-PET biomarkers in DLB and improve clinical practices in diagnosing and managing DLB from an investigational standpoint. Furthermore, this study stresses the importance of identifying the primary visual and visual association cortices in patients with suspected Dementia with Lewy Bodies let alone any dementia syndrome to begin with (Figure 1).

## Conclusions

- Occipital hypometabolism as an *in vivo* biomarker was found to be grossly under-reported on clinical reports.
- Our data suggest the occipital hypometabolism might be missed clinically but the frequency is actually higher than previously reported.
- Future directions would include reproducing these data using large datasets and comparing the central reads with the local reads. Also, a retrospective analysis of existing imaging datasets to confirm these findings would be of interest.

## Figures and Tables

**Table 1:** Demographic characteristics of the patients analyzed retrospectively in this study.

**Age (y)**	73.77 ± 7.95
**Male:Female**	23:12
**Disease Duration (y)**	3.71 ± 2.42
**Fluctuating Cognition**	94.29%
**Visual Hallucinations**	57.14%
**Parkinsonism**	88.57%
**Definite DLB**	17 (48.57%)
**Probable DLB**	16 (45.71%)
**Possible DLB**	2 (5.71%)

**Table 2:** Percent prevalence for hypometabolism of different regions of interest in the above patient cohort on radiology reports and independent read.

Region of Interest	Prevalence in Radiology Reports	Prevalence on Independent Read
Frontal	37.14% (n= 13)	42.86% (n= 15)
Temporal	88.57% (n=31)	85.71% (n= 30)
Parietal	91.43% (n= 32)	91.43% (n= 32)
Occipital	25.71% (n= 9)	91.43% (n= 32)
Cingulate	17.14% (n= 6)	85.71% (n= 30)

**Table 3:** Percent prevalence for occipital hypometabolism across definite, probable, and possible DLB.

DLB per McKeith Consensus Criteria	% Occipital Hypometabolism in Reports	% Occipital Hypometabolism on Independent Reads
Definite DLB	23.53% (n= 4/17)	94.1% (n= 16/17)
Probable DLB	25% (n= 4/16)	93.8% (n= 15/16)
Possible DLB	50% (n= 1/2)	100% (n= 2/2)

**Table 4:** Summary of kappa measure of agreement between radiology reports and independent reads.

Region of Interest	Cohen’s Kappa Statistic	p-value
Frontal	−0.068	0.686
Temporal	0.109	0.515
Parietal	0.635	0.000
Occipital	0.063	0.287
Cingulate	0.067	0.272

## References

[1] Donaghy PC, McKeith IG (2014). The clinical characteristics of dementia with lewy bodies and a consideration of prodromal diagnosis. Alzheimers Res Ther.

[2] McKeith IG, Boeve BF, Dickson DW, Halliday G, Taylor JP (2017). Diagnosis and management of dementia with Lewy bodies: Fourth consensus report of the DLB Consortium. Neurology.

[3] Ishii K (2014). PET Approaches for Diagnosis of Dementia. AJNR Am J Neuroradiol.

[4] Albin RL, Minoshima S, D’Amato CJ, Frey A, Kuhl A (1996). Fluoro-deoxyglucose positron emission tomography in diffuse lewy body disease. Neurology.

[5] Ishii K, Imamura T, Sasaki M, Yamaji S, Sakamoto S (1998). Regional cerebral glucose metabolism in dementia with lewy bodies and Alzheimer’s disease. Neurology.

[6] Minoshima S, Foster NL, Sima AA, Frey KA, Albin RL (2001). Alzheimer’s disease versus dementia with Lewy bodies: Cerebral metabolic distinction with autopsy confirmation. Ann Neurol.

[7] Mosconi L, Tsui WH, Herholz K, Pupi A, Drzezga A (2008). Multicenter standardized 18F-FDG PET diagnosis of mild cognitive impairment, Alzheimer’s disease, and other dementias. J Nucl Med.

[8] O’Brien JT, Firbank MJ, Davison C, Barnett N, Bamford C (2014). 18F-FDG PET and Perfusion SPECT in the diagnosis of Alzheimer and lewy body dementias. J Nucl Med.

[9] Fujishiro H, Iseki E, Kasanuki K, Murayama N, Ota K (2012). Glucose hypometabolism in primary visual cortex is commonly associated with clinical features of dementia with Lewy bodies regardless of cognitive conditions. Int J Geriatr Psychiatry.

[10] Lim SM, Katsifis A, Villemagne VL, Best R, Jones G (2009). The 18F-FDG PET cingulate island sign and comparison to 123I-beta-CIT SPECT for diagnosis of dementia with lewy bodies. J Nucl Med.

[11] Prosser AMJ, Tossici-Bolt L, Kipps CM (2017). Occipital lobe and posterior cingulate perfusion in the prediction of dementia with Lewy body pathology in a clinical sample. Nucl Med Commun.

[12] Graff-Radford J, Murray ME, Lowe VJ, Boeve BF, Ferman TJ (2014). Dementia with lewy bodies: Basis of cingulate island sign. Neurology.

[R13] 13apps.who.int/classifications/icd10/browse/2016/en.

[14] Viera AJ, Garrett JM (2005). Understanding interobserver agreement: The kappa statistic. Family Medicine.

[15] Khundakar AA, Hanson PS, Erskine D, Lax NZ, Roscamp J (2016). Analysis of primary visual cortex in dementia with Lewy Bodies indicates GABAergic involvement associated with recurrent complex visual hallucinations. Acta Neuropathol Commun.

[16] Brown RKJ, Bohnen NI, Wong KK, Minoshima S, Frey KA (2013). Brain PET in suspected dementia: Patterns of altered fdg metabolism. Radiographics.

